# Supplementary material for: Zika virus-induced TNF-α signaling dysregulates expression of neurologic genes associated with psychiatric disorders
Source: J Neuroinflammation. 2022 Apr 24;19:100. doi: 10.1186/s12974-022-02460-8 (PMC9036774; doi:10.1186/s12974-022-02460-8)
Supplement: Supplementary file 1 — Additional file 1: Table S1. Primer sequences for qRT-PCR. Table S2. Curated psychiatric disorder-associated gene list. [file 12974_2022_2460_MOESM1_ESM.pdf]

Additional File 1

Table S1
Primer Sequences for qRT-PCR

Gene	Direction	Sequence
Adora2a	Forward	CACGCAGAGTTCCATCTTCAGC
Adora2a	Reverse	CCCAGCAAATCGCAATGATGCC
Aldh1a2	Forward	CACAAGACACGAGCCCATTGGA
Aldh1a2	Reverse	GGTTTGATGACCACGGTGTTACC
Cacna1e	Forward	ATGGACAAGGCTACCACGGAGA
Cacna1e	Reverse	GACTGGCTTCTCCATCCGTCTT
Ccl12	Forward	GCTACAGGAGAATCACAAGCAGC
Ccl12	Reverse	ACGTCTTATCCAAGTGGTTTATGG
Ccl2	Forward	TGGCTCAGCCAGATGCAGT
Ccl2	Reverse	TTGGGATCATCTTGCTGGTG
Ccl5	Forward	CCTGCTGCTTTGCCTACCTCTC
Ccl5	Reverse	ACACACTTGGCGGTTCCTTCGA
Cd69	Forward	TTTGGAGGGGTTTCAGTGGT
Cd69	Reverse	GCTGTCTACACGGAGGAAGT
Chac1	Forward	TGACCCTCCTTGAAGACCGTGA
Chac1	Reverse	AGTGTCATAGCCACCAAGCACG
Cpne6	Forward	TCAGCTTCACGGTGGCTATCGA
Cpne6	Reverse	CTGTCATAGTCCTGGCAGATACC
Dpp6	Forward	CAATCCTGACCCTCTGTGATGC
Dpp6	Reverse	GCCATCTTTGGAGAACACAGGC
Gpr84	Forward	AAGCCTTCCAGAAGTGCATCGC
Gpr84	Reverse	CAGAGGAACACTGCGAAGCACA
Icam1	Forward	AAACCAGACCCTGGAACTGCAC
Icam1	Reverse	GCCTGGCATTTCAGAGTCTGCT
Ifna6	Forward	CTTCCACAGGATCACTGTGTACCT
Ifna6	Reverse	TTCTGCTCTGACCACCTCCC
Ifnb	Forward	CTGGAGCAGCTGAATGGAAAG
Ifnb	Reverse	CTT CTC CGT CAT CTC CAT AGG G
Ifng	Forward	AACGCTACACACTGCATCTTGG
Ifng	Reverse	GCCGTGGCAGTAACAGCC
Il1b	Forward	ACCTGTCCTGTGTAATGAAAGACG
Il1b	Reverse	TGGGTATTGCTTGGGATCCA
Il6	Forward	CAGAATTGCCATCGTACAACTCTTTTCTCA
Il6	Reverse	AAGGTCATCATCGTTGTTCATACA
Itgb3	Forward	GTGAGTGCGATGACTTCTCCTG
Itgb3	Reverse	CAGGTGTCAGTGCGTGTAGTAC

Lepr	Forward	CTTTCCTGTGGACAGAACCAGC
Lepr	Reverse	AGCACTGAGTGACTCCACAGCA
Lhx6	Forward	AGAGAAAGCACCTCCCAGAA
Lhx6	Reverse	TGGAGTTCTGAACCAAACCA
Met	Forward	GTTCTGCTTGGCAACGAGAGCT
Met	Reverse	GGAGAATGCACTGTATTGCGTCG
Myt1l	Forward	TGTCTGGATGCCCGCACAAAGA
Mytl1	Reverse	CTTCTGTGCGAGTTCCTGTTGC
Nlgn1	Forward	TGATGGGAGTGTCTTGGCAAGC
Nlgn1	Reverse	CCGTAGTTTCCTTTGGCAGCCT
Nr4a2	Forward	GCATACAGGTCCAACCCAGT
Nr4a2	Reverse	AATGCAGGAGAAGGCAGAAA
Penk	Forward	TGCAGCCAGGACTGCGCTAAAT
Penk	Reverse	GATCCTTGCAGGTCTCCCAGAT
Pmch	Forward	CTCTGGAACAATACAAAAACGACG
Pmch	Reverse	GGTTTTACAGCCAGACTCAGTGG
Parb	Forward	GCTTCGTTTGCCAGGACAAGTC
Parb	Reverse	TGGCATCGGTTCCTAGTGACCT
Stat1	Forward	GAACGCGCTCTGCTCAA
Stat1	Reverse	TGCGAATAATATCTGGGAAAGTAA
Syn3	Forward	CCATCTCTGGAAACTGGAAGGC
Syn3	Reverse	CGCCGAACATTTCTGAGCAGCT
Tac1	Forward	TAATGGGCAAGCGGGATGCTGA
Tac1	Reverse	CCATTAGTCCAACAAAGGAATCTG
Tnfa	Forward	AAAATTCGAGTGACAAGCCTGTAGC
Tnfa	Reverse	GTGGGTGAGGAGCACGTAG
Tnfsrsf1a	Forward	ACGAATCACTCTGCTCCGTG
Tnfsrsf1a	Reverse	TCGCAAGGTCTGCATTGTCA
Tnfsrsf1b	Forward	GAGATGCCAAGGTGCCTCAT
Tnfsrsf1b	Reverse	AACTGGGTGCTGTGGTCAAC
Tor3a	Forward	GGTGCCTATGTCTCAGCCTCTT
Tor3a	Reverse	GCCATAGAACTGATGAGAGGGAG

Table S2

Curated Psychiatric Disorder-Associated Gene List

Schizo-phrenia	Bipolar Disorder	Major Depressive Disorder	ADHD	Autism Spectrum Disorder	Implicated gene	Reference(s)
			X		*5HT1B*	(Hawi et al., 2005)
		X			*5HTT*	(Lohoff, 2010)
				X	*ACHE*	(Abrahams et al., 2013)
			X	X	*ACTB*	(Mill et al., 2005), (Zamanian Azodi et al., 2019)
				X	*ACTL6B*	(Abrahams et al., 2013)
				X	*ACY1*	(Abrahams et al., 2013)
				X	*ADA*	(Abrahams et al., 2013)
	X				*ADCY2*	(Mühleisen et al., 2014)
				X	*ADCY3*	(Abrahams et al., 2013)
				X	*ADNP*	(Abrahams et al., 2013)
			X		*ADRA1A*	(Hayman and Fernandez, 2018)
			X		*ADRA1B*	(Hayman and Fernandez, 2018)
				X	*ADSL*	(Abrahams et al., 2013)
				X	*AFF2*	(Abrahams et al., 2013)
				X	*AGAP2*	(Abrahams et al., 2013)
				X	*AGO1*	(Abrahams et al., 2013)
				X	*AGO2*	(Abrahams et al., 2013)
				X	*AGO4*	(Abrahams et al., 2013)
				X	*AHDC1*	(Abrahams et al., 2013)
				X	*AHI1*	(Abrahams et al., 2013)
				X	*AHNAK*	(Abrahams et al., 2013)
				X	*AKAP9*	(Abrahams et al., 2013)
				X	*ALDH1A3*	(Abrahams et al., 2013)
				X	*ALDH5A1*	(Abrahams et al., 2013)
X					*ALDOA*	(Schmidt-Kastner et al., 2020)
				X	*ALG6*	(Abrahams et al., 2013)
X					*ALPK3*	(Schmidt-Kastner et al., 2020)
				X	*AMPD1*	(Abrahams et al., 2013)
	X				*AMPD3*	(Huang et al., 2010)
				X	*AMT*	(Abrahams et al., 2013)
				X	*ANK2*	(Abrahams et al., 2013)
				X	*ANK3*	(Abrahams et al., 2013)
			X	X	*ANKRD11*	(Abrahams et al., 2013)

				X	*ANXA1*	(Abrahams et al., 2013)
				X	*AP1S2*	(Abrahams et al., 2013)
				X	*AP2S1*	(Abrahams et al., 2013)
				X	*APBB1*	(Abrahams et al., 2013)
				X	*APH1A*	(Abrahams et al., 2013)
		X			*APOE*	(Lohoff, 2010)
				X	*ARHGEF9*	(Abrahams et al., 2013)
				X	*ARID1B*	(Abrahams et al., 2013)
			X		*ARTN*	(Demontis et al., 2019)
X					*ARVCF*	(Liu et al., 2019)
				X	*ARX*	(Abrahams et al., 2013)
				X	*ASAP2*	(Abrahams et al., 2013)
				X	*ASH1L*	(Abrahams et al., 2013)
				X	*ASPM*	(Abrahams et al., 2013)
				X	*ASTN2*	(Abrahams et al., 2013)
				X	*ASXL3*	(Abrahams et al., 2013)
X					*ATK3*	(Schmidt-Kastner et al., 2020)
			X		*ATM*	(Hayman and Fernandez, 2018)
				X	*ATP10A*	(Abrahams et al., 2013)
X					*ATP2A2*	(Schmidt-Kastner et al., 2020)
				X	*ATP2B2*	(Abrahams et al., 2013)
			X		*ATP6V0B*	(Demontis et al., 2019)
				X	*ATRX*	(Abrahams et al., 2013)
	X				*ATXN7L3*	(Stahl et al., 2019)
				X	*AUTS2*	(Abrahams et al., 2013)
				X	*AVPR1A*	(Abrahams et al., 2013)
				X	*B4GALT2*	(Demontis et al., 2019)
				X	*BAZ2B*	(Abrahams et al., 2013)
				X	*BCKDK*	(Abrahams et al., 2013)
				X	*BCL11A*	(Abrahams et al., 2013)
				X	*BCORL1*	(Abrahams et al., 2013)
	X	X			*BDNF*	(Lohoff, 2010)
				X	*BICRA*	(Abrahams et al., 2013)
X					*BNIP3L*	(Schmidt-Kastner et al., 2020)
				X	*BRAF*	(Abrahams et al., 2013)
				X	*BRSK2*	(Abrahams et al., 2013)
				X	*BRWD3*	(Abrahams et al., 2013)

				X	*BTAF1*	(Abrahams et al., 2013)
X					*BTG1*	(Schmidt-Kastner et al., 2020)
				X	*C12orf57*	(Abrahams et al., 2013)
				X	*CACNA1A*	(Abrahams et al., 2013)
X	X			X	*CACNA1C*	(Abrahams et al., 2013), (Bhat et al., 2012), (Charney et al., 2017), (Yoshikawa et al., 2021)
				X	*CACNA1D*	(Abrahams et al., 2013)
				X	*CACNA1E*	(Abrahams et al., 2013)
				X	*CACNA1H*	(Abrahams et al., 2013)
				X	*CACNA2D3*	(Abrahams et al., 2013)
	X			X	*Cacna2d4*	(Schmidt-Kastner et al., 2020)
X				X	*CACNB2*	(Abrahams et al., 2013)
				X	*CAMK2B*	(Abrahams et al., 2013)
			X		*CAMK2D*	(Hayman and Fernandez, 2018)
			X		*CAMK2G*	(Hayman and Fernandez, 2018)
				X	*CAPRIN1*	(Abrahams et al., 2013)
				X	*CASK*	(Abrahams et al., 2013)
				X	*CASZ1*	(Abrahams et al., 2013)
		X			*CATSPER1*	(Stahl et al., 2019)
				X	*CC2D1A*	(Abrahams et al., 2013)
			X		*CCDC24*	(Demontis et al., 2019)
				X	*CCNG1*	(Abrahams et al., 2013)
				X	*CCNK*	(Abrahams et al., 2013)
				X	*CCT4*	(Abrahams et al., 2013)
				X	*CDC42BPB*	(Abrahams et al., 2013)
				X	*CDH13*	(Abrahams et al., 2013)
				X	*Cdh8*	(Pagnamenta et al., 2011)
				X	*CDK13*	(Abrahams et al., 2013)
X					*CDK2AP1*	(Schmidt-Kastner et al., 2020)
				X	*CDK8*	(Abrahams et al., 2013)
				X	*CDKL5*	(Abrahams et al., 2013)
				X	*CELF4*	(Abrahams et al., 2013)
				X	*CEP135*	(Abrahams et al., 2013)
				X	*CEP41*	(Abrahams et al., 2013)
				X	*CGNL1*	(Abrahams et al., 2013)
				X	*CHAMP1*	(Abrahams et al., 2013)
				X	*CHD2*	(Abrahams et al., 2013)

				X	*CHD3*	(Abrahams et al., 2013)
				X	*CHD7*	(Abrahams et al., 2013)
				X	*CHD8*	(Abrahams et al., 2013)
				X	*CHKB*	(Abrahams et al., 2013)
				X	*CHMP1A*	(Abrahams et al., 2013)
				X	*CHRNA7*	(Abrahams et al., 2013)
				X	*CIB2*	(Abrahams et al., 2013)
				X	*CIC*	(Abrahams et al., 2013)
				X	*CLASP1*	(Abrahams et al., 2013)
X					*CLCN3*	(Christensen and Børglum, 2019)
X					*CLU*	(Schmidt-Kastner et al., 2020)
				X	*CNKSR2*	(Abrahams et al., 2013)
				X	*CNOT1*	(Abrahams et al., 2013), (Arcos-Burgos et al., 2012)
				X	*CNOT3*	(Abrahams et al., 2013), (Christensen and Børglum, 2019)
			X	X	*CNR1*	(Abrahams et al., 2013)
				X	*CNTN3*	(Chaste and Leboyer, 2012)
X				X	*CNTN4*	(Abrahams et al., 2013)
				X	*CNTN5*	(Abrahams et al., 2013)
				X	*CNTN6*	(Abrahams et al., 2013)
				X	*CNTNAP2*	(Abrahams et al., 2013)
				X	*CNTNAP3*	(Abrahams et al., 2013)
				X	*CNTNAP4*	(Abrahams et al., 2013)
		X	X		*COMT*	(Shadrina et al., 2018)
				X	*CORO1A*	(Abrahams et al., 2013)
X					*CPEB1*	(Schmidt-Kastner et al., 2020)
				X	*CPEB4*	(Abrahams et al., 2013)
X					*CPT1C*	(Schmidt-Kastner et al., 2020)
X					*CREB3L1*	(Schmidt-Kastner et al., 2020)
				X	*CREBBP*	(Abrahams et al., 2013)
				X	*CSDE1*	(Abrahams et al., 2013)
				X	*CSNK2A1*	(Abrahams et al., 2013)
	X				*CSRNP3*	(Stahl et al., 2019)
				X	*CTCF*	(Abrahams et al., 2013)
				X	*CTNNA2*	(Abrahams et al., 2013)
				X	*CTNNB1*	(Abrahams et al., 2013)

				X	*CTNND2*	(Abrahams et al., 2013)
				X	*CTTNBP2*	(Abrahams et al., 2013)
				X	*CUL3*	(Abrahams et al., 2013)
X					*CUL3EP300*	(Schmidt-Kastner et al., 2020)
				X	*CUL7*	(Abrahams et al., 2013)
				X	*CUX1*	(Abrahams et al., 2013)
				X	*CYFIP1*	(Abrahams et al., 2013)
				X	*CYP27A1*	(Abrahams et al., 2013), (Liu et al., 2019)
X					*DAO*	(Verrall et al., 2010)
X		X			*DAOA*	(Arias et al., 2014)
				X	*DAPP1*	(Abrahams et al., 2013)
	X	X	X		*DAT1*	(Hawi et al., 2005)
			X		*DBH*	(Hawi et al., 2005)
				X	*DCC*	(Abrahams et al., 2013)
	X				*DCLK3*	(Chen et al., 2013), (McMahon and Detera-Wadleigh, 2020)
				X	*DDHD2*	(Abrahams et al., 2013)
				X	*DDX3X*	(Abrahams et al., 2013)
				X	*DEAF1*	(Abrahams et al., 2013)
				X	*DENR*	(Abrahams et al., 2013)
				X	*DEPDC5*	(Abrahams et al., 2013)
	X				*DGKH*	(Baum et al., 2008)
				X	*DHCR7*	(Abrahams et al., 2013)
	X				*DHH*	(Charney et al., 2017), (McMahon and Detera-Wadleigh, 2020), (Hou et al., 2016)
				X	*DHX30*	(Abrahams et al., 2013)
				X	*DIP2A*	(Abrahams et al., 2013)
				X	*DIP2C*	(Abrahams et al., 2013)
			X		*DIRAS2*	(Hayman and Fernandez, 2018)
X		X		X	*DISC1*	(Abrahams et al., 2013), (Thomson et al., 2013)
				X	*DLG2*	(Abrahams et al., 2013)
				X	*DLG4*	(Abrahams et al., 2013)
				X	*DLGAP1*	(Abrahams et al., 2013)
				X	*Dlgap2*	(Poquet et al., 2016)
				X	*DLX3*	(Abrahams et al., 2013)
				X	*DMD*	(Abrahams et al., 2013)
				X	*DMPK*	(Abrahams et al., 2013)

	X				*DNAH1*	(McMahon and Detera-Wadleigh, 2020), (Chen et al., 2013)
				X	*DNMT3A*	(Abrahams et al., 2013)
				X	*DOCK8*	(Abrahams et al., 2013)
				X	*DOLK*	(Abrahams et al., 2013)
			X		*DPH2*	(Demontis et al., 2019)
				X	*DPP10*	(Abrahams et al., 2013)
				X	*Dpp6*	(Marshall et al., 2008)
				X	*DPYSL2*	(Abrahams et al., 2013)
	X				*DRD1*	(Ni et al., 2002)
X					*DRD2*	(Beaulieu and Gainetdinov, 2011)
				X	*DRD3*	(Shadrina et al., 2018)
	X	X	X		*DRD4*	(Shadrina et al., 2018)
			X		*DRD5*	(Mill et al., 2005)
				X	*DSCAM*	(Abrahams et al., 2013)
			X		*DUSP6*	(Demontis et al., 2019)
				X	*DYNC1H1*	(Abrahams et al., 2013)
				X	*DYRK1A*	(Abrahams et al., 2013)
				X	*EBF3*	(Abrahams et al., 2013)
				X	*EEF1A2*	(Abrahams et al., 2013)
				X	*EFR3A*	(Abrahams et al., 2013)
				X	*EHMT1*	(Abrahams et al., 2013)
				X	*EIF3G*	(Abrahams et al., 2013)
				X	*ELAVL3*	(Abrahams et al., 2013)
				X	*ELP4*	(Abrahams et al., 2013)
				X	*EMSY*	(Abrahams et al., 2013)
				X	*EP300*	(Abrahams et al., 2013)
				X	*EP400*	(Abrahams et al., 2013)
X					*ERBB4*	(Mei and Xiong, 2008)
				X	*ERBIN*	(Abrahams et al., 2013)
X					*ESRP2*	(Schmidt-Kastner et al., 2020)
				X	*ETFB*	(Abrahams et al., 2013), (Schmidt-Kastner et al., 2020)
				X	*FAM92B*	(Abrahams et al., 2013)
				X	*FBN1*	(Abrahams et al., 2013)
				X	*FBRSL1*	(Abrahams et al., 2013)
X				X	*FGFR1*	(Abrahams et al., 2013), (Demontis et al., 2019)

				X	*FMR1*	(Abrahams et al., 2013)
				X	*FOXG1*	(Abrahams et al., 2013)
				X	*FOXP1*	(Abrahams et al., 2013)
			X	X	*FOXP2*	(Abrahams et al., 2013)
				X	*FRMPD4*	(Abrahams et al., 2013)
	X				*FSTL5*	(Stahl et al., 2019)
X					*FURIN*	(Schmidt-Kastner et al., 2020)
				X	*GABRA3*	(Abrahams et al., 2013)
				X	*GABRB2*	(Abrahams et al., 2013)
				X	*GABRB3*	(Abrahams et al., 2013)
				X	*GABRG3*	(Abrahams et al., 2013)
	X				*GAL3ST3*	(Stahl et al., 2019)
				X	*GALNT10*	(Abrahams et al., 2013)
				X	*GALNT2*	(Abrahams et al., 2013)
	X				*GALNT3*	(Stahl et al., 2019)
				X	*GALNT8*	(Abrahams et al., 2013)
				X	*GATM*	(Abrahams et al., 2013)
				X	*GFAP*	(Abrahams et al., 2013)
				X	*GGNBP2*	(Abrahams et al., 2013)
				X	*GIGYF1*	(Abrahams et al., 2013)
				X	*GIGYF2*	(Abrahams et al., 2013)
			X		*GIT1*	(Palladino et al., 2019)
				X	*GNAI1*	(Abrahams et al., 2013)
X					*GNB3*	(Lohoff, 2010),
				X	*GPC4*	(Abrahams et al., 2013)
				X	*GPHN*	(Abrahams et al., 2013)
	X				*GRB7*	(McMahon and Detera-Wadleigh, 2020), (Hou et al., 2016)
X				X	*GRIA1*	(Abrahams et al., 2013), (Barkus et al., 2014)
				X	*Gria3*	(Chaste and Leboyer, 2012)
				X	*GRID1*	(Abrahams et al., 2013)
				X	*GRIK2*	(Abrahams et al., 2013)
				X	*GRIK5*	(Abrahams et al., 2013)
				X	*GRIN1*	(Abrahams et al., 2013)
X	X			X	*GRIN2A*	(Abrahams et al., 2013)
X			X	X	*GRIN2B*	(Abrahams et al., 2013), (Yoshikawa et al., 2021)

				X	*GRIP1*	(Abrahams et al., 2013)
			X		*GRM1*	(Hayman and Fernandez, 2018)
X	X				*GRM3*	(Kandaswamy et al., 2013)
				X	*HCFC1*	(Abrahams et al., 2013)
				X	*HCN1*	(Abrahams et al., 2013)
				X	*HDAC8*	(Abrahams et al., 2013)
				X	*HDLBP*	(Abrahams et al., 2013)
				X	*HECTD4*	(Abrahams et al., 2013)
				X	*HECW2*	(Abrahams et al., 2013)
				X	*HEPACAM*	(Abrahams et al., 2013)
				X	*HERC2*	(Abrahams et al., 2013)
				X	*HIVEP2*	(Abrahams et al., 2013)
				X	*HIVEP3*	(Abrahams et al., 2013)
				X	*HMGN1*	(Abrahams et al., 2013)
				X	*HNRNPH2*	(Abrahams et al., 2013)
				X	*HNRNPU*	(Abrahams et al., 2013)
				X	*HOXA1*	(Abrahams et al., 2013)
				X	*HRAS*	(Abrahams et al., 2013)
X					*HSPA9*	(Schmidt-Kastner et al., 2020)
X					*HSPD1*	(Schmidt-Kastner et al., 2020)
X					*HSPE1*	(Schmidt-Kastner et al., 2020)
		X			*HTR12A*	(Shadrina et al., 2018)
		X			*HTR1A*	(Shadrina et al., 2018)
		X			*HTR1B*	(Shadrina et al., 2018)
	X	X			*HTR2A*	(Lohoff, 2010)
		X			*HTR2C*	(Shadrina et al., 2018)
	X				*HTTLPR*	(Neves et al., 2008)
				X	*HUWE1*	(Abrahams et al., 2013)
				X	*ICA1*	(Abrahams et al., 2013)
				X	*ILF2*	(Abrahams et al., 2013)
X					*INA*	(Schmidt-Kastner et al., 2020)
				X	*INTS1*	(Abrahams et al., 2013)
				X	*INTS6*	(Abrahams et al., 2013)
			X		*IPO13*	(Demontis et al., 2019)
				X	*IQSEC2*	(Abrahams et al., 2013)
				X	*IRF2BPL*	(Abrahams et al., 2013)
				X	*ITGB3*	(Abrahams et al., 2013)

				X	*JARID2*	(Abrahams et al., 2013)
				X	*KANSL1*	(Abrahams et al., 2013)
				X	*KAT2B*	(Abrahams et al., 2013)
X					*KAT5*	(Schmidt-Kastner et al., 2020)
				X	*KAT6A*	(Abrahams et al., 2013)
				X	*KATNAL2*	(Abrahams et al., 2013)
				X	*KCNB1*	(Abrahams et al., 2013)
				X	*KCNJ10*	(Abrahams et al., 2013)
				X	*KCNQ2*	(Abrahams et al., 2013)
				X	*KCNQ3*	(Abrahams et al., 2013)
				X	*KCNS3*	(Abrahams et al., 2013)
				X	*KDM3B*	(Abrahams et al., 2013)
X			X		*KDM4A*	(Demontis et al., 2019)
				X	*KDM4C*	(Abrahams et al., 2013)
				X	*KDM5B*	(Abrahams et al., 2013)
				X	*KDM5C*	(Abrahams et al., 2013)
				X	*KDM6A*	(Abrahams et al., 2013)
				X	*KDM6B*	(Abrahams et al., 2013)
			X		*KDMA-AS1*	(Demontis et al., 2019)
				X	*KIAA0232*	(Abrahams et al., 2013)
				X	*KIAA1586*	(Abrahams et al., 2013)
				X	*KIF14*	(Abrahams et al., 2013)
				X	*KIRREL3*	(Abrahams et al., 2013)
				X	*KMT2A*	(Abrahams et al., 2013)
				X	*KMT2C*	(Abrahams et al., 2013)
	X				*KMT2D*	(Charney et al., 2017), (McMahon and Detera-Wadleigh, 2020), (Hou et al., 2016)
				X	*KMT2E*	(Abrahams et al., 2013)
				X	*KMT5B*	(Abrahams et al., 2013)
				X	*KPTN*	(Abrahams et al., 2013)
				X	*LAMB1*	(Abrahams et al., 2013)
				X	*LDB1*	(Abrahams et al., 2013)
				X	*LEO1*	(Abrahams et al., 2013)
			X		*LINC00461*	(Demontis et al., 2019)
	X				*LINC01215*	(Stahl et al., 2019)
			X		*LINC01288*	(Demontis et al., 2019)

			X		*LINC01572*	(Demontis et al., 2019)
			X		*LINC02060*	(Demontis et al., 2019)
			X		*LINC02497*	(Demontis et al., 2019)
				X	*LMX1B*	(Abrahams et al., 2013)
				X	*LNPK*	(Abrahams et al., 2013)
	X				*LOC101927314*	(Mühleisen et al., 2014)
	X				*LOC102725191*	(Stahl et al., 2019)
	X				*LOC105371789*	(Stahl et al., 2019)
			X		*LPHN3*	(Schmidt-Kastner et al., 2020)
X				X	*LRP1*	(Abrahams et al., 2013)
				X	*LRRC4C*	(Abrahams et al., 2013)
X					*LSM1*	(Schmidt-Kastner et al., 2020)
				X	*LZTR1*	(Abrahams et al., 2013)
				X	*MACROD2*	(Abrahams et al., 2013)
X					*MAD1L1*	(Schmidt-Kastner et al., 2020)
				X	*MAGEL2*	(Abrahams et al., 2013)
		X			*MAOA*	(Shadrina et al., 2018)
				X	*MAP1A*	(Abrahams et al., 2013)
				X	*MAPT-AS1*	(Abrahams et al., 2013)
				X	*MBD5*	(Abrahams et al., 2013)
				X	*MBOAT7*	(Abrahams et al., 2013)
X					*MDK*	(Schmidt-Kastner et al., 2020)
				X	*MECP2*	(Abrahams et al., 2013)
				X	*MED13*	(Abrahams et al., 2013)
				X	*MED13L*	(Abrahams et al., 2013)
X				X	*MEF2C*	(Abrahams et al., 2013)
				X	*MEIS2*	(Abrahams et al., 2013)
				X	*MET*	(Abrahams et al., 2013)
				X	*MFRP*	(Abrahams et al., 2013)
				X	*MIEN1*	(McMahon and Detera-Wadleigh, 2020), (Hou et al., 2016)
X				X	*MIR137*	(Pasquinelli, 2012)
	X				*MIR3127*	(McMahon and Detera-Wadleigh, 2020), (Chen et al., 2013)
			X		*MIR3666*	(Demontis et al., 2019)
	X				*MIR4655*	(McMahon and Detera-Wadleigh, 2020), (Hou et al., 2016)

	X				*MIR4728*	(McMahon and Detera-Wadleigh, 2020), (Hou et al., 2016)
	X				*MIR640*	(Cichon et al., 2011), (McMahon and Detera-Wadleigh, 2020)
			X		*MIR9-2*	(Demontis et al., 2019)
	X				*MIRLET7G*	(McMahon and Detera-Wadleigh, 2020), (Chen et al., 2013)
				X	*MKX*	(Abrahams et al., 2013)
				X	*MSL3*	(Abrahams et al., 2013)
				X	*MSNP1AS*	(Abrahams et al., 2013)
X		X			*MTHFR*	(Lohoff, 2010)
				X	*MTOR*	(Abrahams et al., 2013)
	X				*MXI1*	(Charney et al., 2017), (McMahon and Detera-Wadleigh, 2020)
				X	*MYH10*	(Abrahams et al., 2013)
				X	*MYH9*	(Abrahams et al., 2013)
				X	*MYO5A*	(Abrahams et al., 2013)
				X	*MYO9B*	(Abrahams et al., 2013)
				X	*MYT1L*	(Abrahams et al., 2013)
				X	*NAA15*	(Abrahams et al., 2013)
				X	*NACC1*	(Abrahams et al., 2013)
				X	*NAV2*	(Abrahams et al., 2013)
				X	*NBEA*	(Abrahams et al., 2013)
	X				*NCAM1*	(Escamilla and Zavala, 2008)
X	X				*NCAN*	(Cichon et al., 2011), (McMahon and Detera-Wadleigh, 2020)
				X	*NCKAP1*	(Abrahams et al., 2013)
				X	*NCOA1*	(Abrahams et al., 2013)
				X	*NCOR1*	(Abrahams et al., 2013)
X					*NDST3*	(Liu et al., 2019)
X					*NEK1*	(Schmidt-Kastner et al., 2020)
				X	*NEXMIF*	(Abrahams et al., 2013)
				X	*NF1*	(Abrahams et al., 2013)
				X	*NFE2L3*	(Abrahams et al., 2013)
	X			X	*NFIX*	(Abrahams et al., 2013)
X					*NGEF*	(Schmidt-Kastner et al., 2020)
X					*NGN2/NEUROG2*	(Christensen and Børglum, 2019)
				X	*NINL*	(Abrahams et al., 2013)

				X	*NIPBL*	(Abrahams et al., 2013)
				X	*NLGN1*	(Abrahams et al., 2013)
				X	*NLGN2*	(Abrahams et al., 2013)
				X	*NLGN3*	(Abrahams et al., 2013)
				X	*NLGN4X*	(Abrahams et al., 2013)
X					*NMB*	(Schmidt-Kastner et al., 2020)
			X		*NOS1*	(Weber et al., 2015)
X					*NOTCH4*	(Liu et al., 2019)
				X	*NOVA2*	(Abrahams et al., 2013)
		X			*NR3C1*	(Lohoff, 2010)
				X	*NR3C2*	(Abrahams et al., 2013)
X		X		X	*NR4A2*	(Abrahams et al., 2013)
X	X				*NRG1*	(Mei and Nave, 2014),<br>(Escamilla and Zavala, 2008)
X					*NRGN*	(Schmidt-Kastner et al., 2020)
				X	*NRXN1*	(Abrahams et al., 2013)
				X	*NRXN2*	(Abrahams et al., 2013)
				X	*NRXN3*	(Abrahams et al., 2013)
				X	*NSD1*	(Abrahams et al., 2013)
				X	*NSD2*	(Abrahams et al., 2013)
				X	*NTNG2*	(Abrahams et al., 2013)
				X	*NTRK2*	(Abrahams et al., 2013)
				X	*NUAK1*	(Abrahams et al., 2013)
				X	*NUDCD2*	(Abrahams et al., 2013)
				X	*NUP155*	(Abrahams et al., 2013)
				X	*OCRL*	(Abrahams et al., 2013)
	X				*ODZ4*	(Kerner, 2014)
X					*OGFOD2*	(Schmidt-Kastner et al., 2020)
X					*OPCML*	(Schmidt-Kastner et al., 2020)
				X	*OPHN1*	(Abrahams et al., 2013)
				X	*OR52M1*	(Abrahams et al., 2013)
				X	*OTUD7A*	(Abrahams et al., 2013)
X	X				*OTUD7B*	(Schmidt-Kastner et al., 2020)
				X	*OXTR*	(Abrahams et al., 2013)
				X	*P2RX5*	(Abrahams et al., 2013)
				X	*P4HA2*	(Abrahams et al., 2013)
				X	*PACS1*	(Abrahams et al., 2013)

				X	*PACS2*	(Abrahams et al., 2013)
				X	*PAH*	(Abrahams et al., 2013)
				X	*PAK1*	(Abrahams et al., 2013)
				X	*PAK2*	(Abrahams et al., 2013)
				X	*PARD3B*	(Abrahams et al., 2013)
			X		*PARK2*	(Hayman and Fernandez, 2018)
				X	*PAX5*	(Abrahams et al., 2013)
X				X	*PAX6*	(Abrahams et al., 2013), (Yoshikawa et al., 2021)
				X	*PCCA*	(Abrahams et al., 2013)
				X	*PCCB*	(Abrahams et al., 2013)
				X	*PCDH19*	(Abrahams et al., 2013)
				X	*PCDH7*	(Demontis et al., 2019)
				X	*Pcdh9*	(Marshall et al., 2008)
		X			*PCLO*	(Shadrina et al., 2018)
	X				*PDE10A*	(Kerner et al., 2011)
X					*PDE4B*	(Liu et al., 2019)
			X		*PDE4D*	(Hayman and Fernandez, 2018)
				X	*PER2*	(Abrahams et al., 2013)
X					*PGBD1*	(Liu et al., 2019)
X					*PGM3*	(Schmidt-Kastner et al., 2020)
				X	*PHB*	(Abrahams et al., 2013)
				X	*PHF12*	(Abrahams et al., 2013)
				X	*PHF2*	(Abrahams et al., 2013)
				X	*PHF21A*	(Abrahams et al., 2013)
				X	*PHF3*	(Abrahams et al., 2013)
				X	*PHF7*	(Abrahams et al., 2013)
				X	*PHF8*	(Abrahams et al., 2013)
				X	*PHIP*	(Abrahams et al., 2013)
				X	*PHRF1*	(Abrahams et al., 2013)
				X	*PIK3R2*	(Abrahams et al., 2013)
				X	*PLCB1*	(Abrahams et al., 2013)
				X	*PLXNA4*	(Abrahams et al., 2013)
				X	*PLXNB1*	(Abrahams et al., 2013)
			X		*POC1B*	(Demontis et al., 2019)
				X	*POGZ*	(Abrahams et al., 2013)
				X	*POMGNT1*	(Abrahams et al., 2013)

				X	*PON1*	(Abrahams et al., 2013)
				X	*POU3F3*	(Abrahams et al., 2013)
				X	*PPP1R9B*	(Abrahams et al., 2013)
				X	*PPP2CA*	(Abrahams et al., 2013)
X					*PPP2R2A*	(Schmidt-Kastner et al., 2020)
				X	*PPP2R5D*	(Abrahams et al., 2013)
				X	*PPP5C*	(Abrahams et al., 2013)
				X	*PREX1*	(Abrahams et al., 2013)
				X	*PRICKLE1*	(Abrahams et al., 2013)
				X	*PRICKLE2*	(Abrahams et al., 2013)
				X	*PRKCB*	(Abrahams et al., 2013)
				X	*PRKD1*	(Abrahams et al., 2013)
				X	*PRKD2*	(Abrahams et al., 2013)
				X	*PRKN*	(Abrahams et al., 2013)
X					*PRMT1*	(Schmidt-Kastner et al., 2020)
				X	*PRODH*	(Abrahams et al., 2013)
				X	*PRR12*	(Abrahams et al., 2013)
X					*PRSS16/POM121L2*	(Liu et al., 2019)
				X	*PSMD12*	(Abrahams et al., 2013)
				X	*PTCHD1*	(Abrahams et al., 2013)
				X	*PTCHD1-AS*	(Abrahams et al., 2013)
				X	*PTEN*	(Abrahams et al., 2013)
X					*PTK2B*	(Schmidt-Kastner et al., 2020)
				X	*PTK7*	(Abrahams et al., 2013)
				X	*PTPN11*	(Abrahams et al., 2013)
			X		*PTPRF*	(Demontis et al., 2019)
				X	*PYHIN1*	(Abrahams et al., 2013)
				X	*QRICH1*	(Abrahams et al., 2013)
				X	*RAB2A*	(Abrahams et al., 2013)
				X	*RAB43*	(Abrahams et al., 2013)
				X	*RAC1*	(Abrahams et al., 2013)
				X	*RAD21*	(Abrahams et al., 2013)
			X	X	*RAI1*	(Abrahams et al., 2013)
				X	*RALA*	(Abrahams et al., 2013)
X					*RALGAPA2*	(Schmidt-Kastner et al., 2020)
				X	*RALGAPB*	(Abrahams et al., 2013)

				X	*RANBP17*	(Abrahams et al., 2013)
X					*RANGAP1*	(Schmidt-Kastner et al., 2020)
				X	*RBFOX1*	(Abrahams et al., 2013)
				X	*RBM27*	(Abrahams et al., 2013)
	X				*RBPJL*	(Stahl et al., 2019)
X					*RELA*	(Schmidt-Kastner et al., 2020)
				X	*RELN*	(Abrahams et al., 2013)
				X	*RERE*	(Abrahams et al., 2013)
				X	*RFX3*	(Abrahams et al., 2013)
				X	*RHEB*	(Abrahams et al., 2013)
				X	*RHEBL1*	(Charney et al., 2017), (McMahon and Detera-Wadleigh, 2020), (Hou et al., 2016)
				X	*RIMS1*	(Abrahams et al., 2013)
				X	*RLIM*	(Abrahams et al., 2013)
	X				*RNA5SP223*	(McMahon and Detera-Wadleigh, 2020)
	X				*RNU2-17P*	(Stahl et al., 2019)
	X				*RNU6-1028P*	(Cichon et al., 2011), (McMahon and Detera-Wadleigh, 2020)
	X				*RNU6-131P*	(Stahl et al., 2019)
				X	*ROBO2*	(Abrahams et al., 2013)
				X	*RORA*	(Abrahams et al., 2013)
				X	*RORB*	(Abrahams et al., 2013)
				X	*RPS10P2-AS1*	(Abrahams et al., 2013)
	X				*RPS6KA2*	(Kerner et al., 2011)
X					*RPTOR*	(Schmidt-Kastner et al., 2020)
				X	*RSRC1*	(Abrahams et al., 2013)
				X	*SAE1*	(Abrahams et al., 2013)
				X	*SATB1*	(Abrahams et al., 2013)
				X	*SBF1*	(Abrahams et al., 2013)
				X	*SCAF4*	(Abrahams et al., 2013)
				X	*SCN1A*	(Abrahams et al., 2013)
				X	*SCN2A*	(Abrahams et al., 2013)
	X				*SCN2A*	(Stahl et al., 2019)
				X	*SCN8A*	(Abrahams et al., 2013)
				X	*SCN9A*	(Abrahams et al., 2013)
				X	*SEMA5A*	(Abrahams et al., 2013)
			X		*SEMA6D*	(Demontis et al., 2019)

X					*SERPING1*	(Schmidt-Kastner et al., 2020)
				X	*SET*	(Abrahams et al., 2013)
				X	*SETBP1*	(Abrahams et al., 2013)
				X	*SETD1A*	(Abrahams et al., 2013)
				X	*SETD2*	(Abrahams et al., 2013)
				X	*SETD5*	(Abrahams et al., 2013)
X					*SF3B1*	(Schmidt-Kastner et al., 2020)
				X	*SGSH*	(Abrahams et al., 2013)
				X	*SHANK1*	(Abrahams et al., 2013)
X	X			X	*SHANK2*	(Abrahams et al., 2013), (Yoshikawa et al., 2021)
				X	*SHANK3*	(Abrahams et al., 2013)
				X	*SHOX*	(Abrahams et al., 2013)
				X	*SIK1*	(Abrahams et al., 2013)
				X	*SIN3A*	(Abrahams et al., 2013)
				X	*SKI*	(Abrahams et al., 2013)
				X	*SLC12A5*	(Abrahams et al., 2013)
				X	*SLC1A2*	(Abrahams et al., 2013)
				X	*SLC35B1*	(Abrahams et al., 2013)
				X	*SLC38A10*	(Abrahams et al., 2013)
				X	*SLC45A1*	(Abrahams et al., 2013)
				X	*SLC6A1*	(Abrahams et al., 2013)
		X			*SLC6A2*	(Lohoff, 2010)
		X	X	X	*SLC6A3*	(Abrahams et al., 2013)
	X	X	X		*SLC6A4*	(Escamilla and Zavala, 2008)
			X		*SLC6A9*	(Demontis et al., 2019)
				X	*SLC7A3*	(Abrahams et al., 2013)
				X	*SLC7A5*	(Abrahams et al., 2013)
				X	*SLC9A6*	(Abrahams et al., 2013)
				X	*SLITRK5*	(Abrahams et al., 2013)
				X	*SMAD4*	(Abrahams et al., 2013)
				X	*SMARCA2*	(Abrahams et al., 2013)
				X	*SMARCA4*	(Abrahams et al., 2013)
				X	*SMARCC2*	(Abrahams et al., 2013)
				X	*SMC1A*	(Abrahams et al., 2013)
	X				*SMNDC1*	(Charney et al., 2017), (McMahon and Detera-Wadleigh, 2020)
			X		*SNAP25*	(Mill et al., 2005)

X					*SNAP91*	(Christensen and Børglum, 2019)
				X	*SNX14*	(Abrahams et al., 2013)
				X	*SNX5*	(Abrahams et al., 2013)
				X	*SON*	(Abrahams et al., 2013)
	X				*SORCS1*	(Charney et al., 2017), (McMahon and Detera-Wadleigh, 2020)
			X	X	*SORCS3*	(Abrahams et al., 2013)
				X	*SOX5*	(Abrahams et al., 2013)
				X	*SOX6*	(Abrahams et al., 2013)
			X		*SPAG16*	(Demontis et al., 2019)
				X	*SPARCL1*	(Abrahams et al., 2013)
				X	*SPAST*	(Abrahams et al., 2013)
				X	*SPEN*	(Abrahams et al., 2013)
				X	*SRCAP*	(Abrahams et al., 2013)
X					*SREBF1*	(Schmidt-Kastner et al., 2020)
X					*SRPK2*	(Schmidt-Kastner et al., 2020)
				X	*SRPRA*	(Abrahams et al., 2013)
X					*SRR*	(Balu et al., 2013)
				X	*SRSF11*	(Abrahams et al., 2013)
			X		*ST3GAL3*	(Demontis et al., 2019)
				X	*ST8SIA2*	(Abrahams et al., 2013)
				X	*STAG1*	(Abrahams et al., 2013)
				X	*STXBP1*	(Abrahams et al., 2013)
				X	*STXBP5*	(Abrahams et al., 2013)
				X	*SUPT16H*	(Abrahams et al., 2013)
				X	*SYN1*	(Abrahams et al., 2013)
X					*Syn3*	(Chen et al., 2009)
	X				*SYNE1*	(McMahon and Detera-Wadleigh, 2020)
	X				*SYNE1-AS1*	(McMahon and Detera-Wadleigh, 2020)
				X	*SYNGAP1*	(Abrahams et al., 2013)
				X	*SYT1*	(Abrahams et al., 2013)
			X		*SYT2*	(Hayman and Fernandez, 2018)
				X	*TAF1*	(Abrahams et al., 2013)
				X	*TAF6*	(Abrahams et al., 2013)
				X	*TANC2*	(Abrahams et al., 2013)
				X	*TAOK1*	(Abrahams et al., 2013)

				X	*TAOK2*	(Abrahams et al., 2013)
				X	*TBC1D23*	(Abrahams et al., 2013)
				X	*TBC1D31*	(Abrahams et al., 2013)
				X	*TBCK*	(Abrahams et al., 2013)
				X	*TBL1XR1*	(Abrahams et al., 2013)
				X	*TBR1*	(Abrahams et al., 2013)
				X	*TBX1*	(Abrahams et al., 2013)
				X	*TCF20*	(Abrahams et al., 2013)
X				X	*TCF4*	(Abrahams et al., 2013)
				X	*TCF7L2*	(Abrahams et al., 2013)
				X	*TEK*	(Abrahams et al., 2013)
				X	*TERF2*	(Abrahams et al., 2013)
				X	*TET2*	(Abrahams et al., 2013)
				X	*TET3*	(Abrahams et al., 2013)
		X			*TH*	(Shadrina et al., 2018)
	X				*THSD7A*	(Stahl et al., 2019)
				X	*TLK2*	(Abrahams et al., 2013)
	X				*TLR9*	(Chen et al., 2013), (McMahon and Detera-Wadleigh, 2020)
				X	*TM4SF20*	(Abrahams et al., 2013)
				X	*TM9SF4*	(Abrahams et al., 2013)
	X				*TMEM151A*	(Stahl et al., 2019)
				X	*TMLHE*	(Abrahams et al., 2013)
	X				*TMUB2*	(Stahl et al., 2019)
				X	*TNRC6B*	(Abrahams et al., 2013)
				X	*TOR3A*	(LeDoux, 2012)
		X	X		*TPH1*	(Palladino et al., 2019)
		X	X		*TPH2*	(Palladino et al., 2019)
				X	*TRAF7*	(Abrahams et al., 2013)
	X				*TRANK1*	(Chen et al., 2013), (McMahon and Detera-Wadleigh, 2020)
				X	*TRAPPC6B*	(Abrahams et al., 2013)
				X	*TRAPPC9*	(Abrahams et al., 2013)
				X	*TRIM23*	(Abrahams et al., 2013)
				X	*TRIO*	(Abrahams et al., 2013)
				X	*TRIP12*	(Abrahams et al., 2013)
				X	*TRPC6*	(Abrahams et al., 2013)
				X	*TRPM1*	(Abrahams et al., 2013)

				X	*TSC1*	(Abrahams et al., 2013)
				X	*TSC2*	(Abrahams et al., 2013)
				X	*TSHZ3*	(Abrahams et al., 2013)
X					*TSNARE1*	(Christensen and Børglum, 2019)
				X	*TTI2*	(Abrahams et al., 2013)
				X	*UBE3A*	(Abrahams et al., 2013)
				X	*UBE3C*	(Abrahams et al., 2013)
				X	*UBN2*	(Abrahams et al., 2013)
				X	*UBR1*	(Abrahams et al., 2013)
				X	*UBR5*	(Abrahams et al., 2013)
				X	*UNC79*	(Abrahams et al., 2013)
				X	*UPF3B*	(Abrahams et al., 2013)
				X	*USP15*	(Abrahams et al., 2013)
				X	*USP45*	(Abrahams et al., 2013)
				X	*USP7*	(Abrahams et al., 2013)
				X	*USP9X*	(Abrahams et al., 2013)
				X	*VAMP2*	(Abrahams et al., 2013)
				X	*VEZF1*	(Abrahams et al., 2013)
				X	*VIL1*	(Abrahams et al., 2013)
				X	*VPS13B*	(Abrahams et al., 2013)
				X	*WAC*	(Abrahams et al., 2013)
				X	*WASF1*	(Abrahams et al., 2013)
				X	*WDFY3*	(Abrahams et al., 2013)
				X	*WDFY4*	(Abrahams et al., 2013)
				X	*WDR26*	(Abrahams et al., 2013)
				X	*WFDC5*	(Stahl et al., 2019)
				X	*WWOX*	(Abrahams et al., 2013)
				X	*XPC*	(Abrahams et al., 2013)
				X	*YY1*	(Abrahams et al., 2013)
				X	*ZBTB20*	(Abrahams et al., 2013)
				X	*ZC3H4*	(Abrahams et al., 2013)
	X				*ZCCHC2*	(Stahl et al., 2019)
X					*ZEB2*	(Schmidt-Kastner et al., 2020)
				X	*ZMIZ1*	(Abrahams et al., 2013)
				X	*ZMYM2*	(Abrahams et al., 2013)
				X	*ZMYND11*	(Abrahams et al., 2013)

				X	*ZMYND8*	(Abrahams et al., 2013)
				X	*ZNF292*	(Abrahams et al., 2013)
				X	*ZNF462*	(Abrahams et al., 2013)
X				X	*ZNF804A*	(Abrahams et al., 2013)
				X	*ZSWIM6*	(Abrahams et al., 2013)
